# Persistent and changing job strain and risk of coronary heart disease. A population-based cohort study of 1.6 million employees in Denmark

**DOI:** 10.5271/sjweh.3891

**Published:** 2020-09-01

**Authors:** Reiner Rugulies, Elisabeth Framke, Jeppe Karl Sørensen, Annemette Coop Svane-Petersen, Kristina Alexanderson, Jens Peter Bonde, Kristin Farrants, Esben Meulengracht Flachs, Linda L Magnusson Hanson, Solja T Nyberg, Mika Kivimäki, Ida EH Madsen

**Affiliations:** 1 National Research Centre for the Working Environment, Copenhagen, Denmark; 2 Section of Epidemiology, Department of Public Health, University of Copenhagen, Denmark; 3 Department of Psychology, University of Copenhagen, Denmark; 4 Division of Insurance Medicine, Department of Clinical Neuroscience, Karolinska Institutet, Stockholm, Sweden; 5 Department of Occupational and Environmental Medicine, Bispebjerg and Frederiksberg Hospital, Denmark; 6 Stress Research Institute, Stockholm University, Stockholm, Sweden; 7 Clinicum, Faculty of Medicine, University of Helsinki, Helsinki, Finland; 8 Helsinki Institute of Life Sciences, Helsinki, Finland; 9 Department of Epidemiology and Public Health, University College London, London, UK

**Keywords:** cardiovascular disease, CHD, epidemiology, JEM, job control, job exposure matrix, psychological demand, psychosocial work environment, stress, work stress

## Abstract

**Objectives:**

This study aimed to examine the association between job strain and incident coronary heart disease (CHD) in Denmark, while accounting for changes of job strain.

**Methods:**

We included all employees residing in Denmark in 2000, aged 30–59 years with no prevalent CHD (N=1 660 150). We determined exposure to job strain from 1996–2009 using a job exposure matrix (JEM) with annual updates. Follow-up for incident CHD was from 2001–2010 via linkage to health records. We used Cox regression to calculate hazard ratios (HR) and 95% confidence intervals (CI) for the association between job strain and incident CHD.

**Results:**

During 16.1 million person-years, we identified 24 159 incident CHD cases (15.0 per 10 000 person-years). After adjustment for covariates, job strain in 2000 predicted onset of CHD during a mean follow-up of 9.71 years (HR 1.10, 95% CI 1.07–1.13). When analyzing changes in job strain from one year to the next and CHD in the subsequent year, persistent job strain (HR 1.07, 95% CI 1.03–1.10), onset of job strain (HR 1.20, 95% CI 1.12–1.29) and removal of strain (HR 1.20, 95% CI 1.12–1.28) were associated with higher CHD incidence compared to persistent no job strain. Associations were similar among men and women.

**Conclusions:**

Job strain is associated with a higher risk of incident CHD in Denmark. As we used a JEM, we can rule out reporting bias. However, under- or overestimation of associations is possible due to non-differential misclassification of job strain and residual confounding by socioeconomic position.

Meta-analyses of prospective cohort studies suggest that adverse psychosocial working conditions may contribute to onset of coronary heart disease (CHD) (1–5). The combination of high psychological demands and low decision latitude at work, denoted “job strain”, has been most extensively studied. An individual participant data (IPD) meta-analysis of 13 European cohort studies showed that job strain was associated with approximately 20% excess risk of CHD [pooled hazard ratio (HR) of 1.23, 95% confidence interval (CI) 1.10–1.37] (1). Another meta-analysis summarizing 31 studies, including the IPD-Work consortium data, showed a slightly higher excess risk (pooled HR 1.33, 95% CI 1.19–1.49) (3). The underlying mechanisms linking job strain to CHD may include activation and dysregulation of the sympatho-adrenal medullary and the hypothalamic–pituitary–adrenal stress axes, inflammatory processes and increase in hazardous health behaviors, such as smoking intensity and leisure-time physical inactivity (6, 7).

However, there are important limitations to the evidence, calling for further research. First, job strain has predominantly been measured by self-reported data, raising concerns about reporting bias. Undetected pre-clinical CHD may cause individuals experiencing work as more strenuous, yielding spurious associations between baseline self-reported job strain and subsequent onset of clinical CHD.

Second, job strain has predominantly been assessed only once, at baseline, and changes in job strain over time usually have not been accounted for, likely resulting in imprecise measurement and exposure misclassification. Furthermore, measuring job strain repeatedly would allow analyzing if job strain over several years is more harmful to health than job strain measured at a single point in time.

Third, it is unknown whether onset of job strain is associated with a higher risk of CHD and that removal of job strain is associated with a lower risk. Identifying such associations would strengthen the interpretation of job strain as a causal factor in the etiology of CHD.

We address these limitations by analyzing the association between job strain and CHD in the Danish workforce while (i) measuring job strain with a job exposure matrix (JEM) (rather than individual-level self-reports) with annual updates, (ii) examining whether number of years with job strain is associated with risk of CHD, and (iii) analyzing the association between persistent job strain, as well as job strain onset and removal, and risk of CHD.

## Methods

### Study design and population

We used data from the JEMPAD (Job Exposure Matrix Analyses of Psychosocial Factors and Healthy Ageing in Denmark) study, a nationwide register-based study on work environment and health. A JEMPAD study on the association between educational attainment and risk of cardiovascular morbidity and mortality, and the role of household income and job strain for this association, was recently published (8).

The study population was drawn from the Integrated Database for Labor Market Research (IDA) at Statistics Denmark (9). We included all individuals residing in Denmark (independent of their nationality), aged 30–59 years and employed (excluding the self-employed) in the year 2000. We excluded 1323 individuals with missing data on age, sex, or migration background, yielding 1 680 214 individuals. Using their unique Danish civil registration number, we linked these individuals to other population-based registers providing information on socio-demographic variables and health.

We excluded 20 064 individuals diagnosed with CHD (ICD 8: 410–414; ICD 10: I20–I25; ICD 9 was never used in Denmark) from 1 January 1977 [when the diagnosis first became available in the National Patient Register (10)] to 31 December 2000 (the day before start of follow-up period), yielding a study population of 1 660 150 individuals. To identify incident CHD during follow-up, we linked these individuals to records from national health registers until 31 December 2010. The duration of follow-up was motivated by keeping the job strain measure consistent across the follow-up period, as this measure was job-group-specific, and the registration of job groups had changed from 2010 onwards.

### Job strain

We estimated the predicted probability of job strain with a JEM based on information from the Danish Work Environment Cohort Study (DWECS) (11, 12). DWECS was a survey on working conditions and health, conducted in a randomly selected sample of the Danish workforce from 1990–2010. We included DWECS data from the years 2000 and 2005 (N=10 749) with information on job strain. In accordance with previous research (1), we measured job strain by combining three items on psychological demands at work and five items on job control, and defined job strain as simultaneously scoring psychological demands above the median and job control below the median. We constructed the JEM in DWECS as the predicted probability of job strain given job group, sex, age, and year of data collection (2000, 2005). Job group was coded according to the four-digit level of DISCO-88, the Danish version of the International Standard Classification of Occupations (ISCO)-88 system (13).

We assigned the predicted probabilities of job strain to each individual from the JEMPAD cohort annually from 1996–2009. We categorized individuals into groups with and without job strain based on the median split of the annual distribution of the predicted probability of job strain. Individuals predominantly non-employed in a given year (eg, due to unemployment, self-employment, disability retirement, or statutory retirement) were assigned a separate category [“not applicable (NA) job strain”] during these years. See the supplementary material (www.sjweh.fi/show_abstract.php?abstract_id=3891), appendix 1 for a detailed description of the JEM.

### Incident coronary heart disease

We ascertained incident CHD by retrieving both main and secondary diagnoses from the National Patient Register (10) and underlying and contributing causes from the Danish Register of Causes of Death (14) from 1 January 2001 to 31 December 2010. The two registers are valid tools for studying CHD at the population level (15). Incident CHD was defined as either incident non-fatal myocardial infarction (ICD-10: I21, I22) or death due to CHD (I20–I25).

### Covariates

As potential confounders, we considered age, sex, migration background, family type, health services use in the year before exposure ascertainment (as an indicator of health status, including possible undiagnosed prevalent CHD) and socioeconomic position, measured by household disposable income. We further presented individuals’ general occupational position (based on the first digit DISCO-88 code) and educational attainment [based on the International Standard Classification of Education (ISCED) (16)] to describe the study population but did not use these two variables for statistical adjustment in the main analyses. Adjusting for occupational position was inadvisable because both occupational position and job strain were based on DISCO-88 codes. Adjusting for education may have resulted in overadjustment because educational attainment is intertwined with job group, as certain levels of education are a necessary prerequisite for entering specific jobs (eg, becoming a lawyer, engineer or a physician requires a university degree). However, not adjusting for education could lead to underadjustment. To get the most complete picture, we therefore reported estimates unadjusted for education in the main analyses and estimates adjusted for education in a supplementary analysis. Further, we conducted separate analyses by level of education to explore if the association between job strain and risk of CHD differed by educational attainment.

Information on age, sex and migration background was retrieved from the Population Register (17). Age was included with piecewise linear splines accounting for nonlinear association between age and CHD (knots at 30–35, 36–42, 43–50 and 51–59 years, respectively). For migration background, we used the classification by Statistics Denmark (17) distinguishing between (i) individuals with no migration background, (ii) immigrants, and (iii) descendants of immigrants. Data on family type was retrieved from the Population Register (17) and the Family Relation Register (18) and categorized into six groups, combining information on marriage/cohabitation with presence of children in the household (see table 1 for details). Information on annual household disposable income, that is the sum of earned income and social transfer payments of all household members after deduction of taxes and interest expenses, was retrieved from registers on personal income and transfer payments (19) and categorized into deciles based on the distribution of individuals within each year. We retrieved information on health services use, provided by primary healthcare professionals, such as general practitioners, from the Danish National Health Service Register (20) and categorized the number of health services used into deciles based on the annual distributions.

**Table 1:** Characteristics of the study population in the year 2000. [ISCED= International Standard Classification of Education; DISCO=Danish version of the International Standard Classification of Occupations; SD=standard deviation.]

	N	%	Mean	SD
Sex				
Men	850 999	51.3		
Women	809 151	48.7		
Age in years			43.70	8.40
Family type				
Single without children	321 053	19.3		
Single with children aged 0–7	27 632	1.66		
Single with children aged 8–17	57 330	3.45		
Married/cohabitant without children	511 283	30.8		
Married/cohabitant with children aged 0–7	244 151	14.7		
Married/cohabitant with children aged 8–17	306 867	18.5		
Missing family type	191 834	11.6		
Migration background				
No migration background	1 585 284	95.5		
Immigrant	72 411	4.4		
Descendant of immigrants	2455	0.2		
Household disposable income (euros)			42 711	36 573
Number of health service used			15.87	20.60
Education				
Low education (ISCED 0–2)	385 815	23.2		
Medium education (ISCED 3–4)	761 455	45.9		
High education (ISCED ≥5)	491 702	29.6		
Missing education	21 178	1.28		
Occupation (based on first DISCO-88 digit)				
Legislators, senior officials, managers	56 152	3.4		
Professionals	252 470	15.2		
Technicians, associate professionals	314 694	19.0		
Clerks	195 051	11.7		
Service workers, shop & market sales workers	191 219	11.5		
Skilled agricultural & fishery workers	5608	0.3		
Craft and related workers	177 772	10.7		
Plant & machine operators, assemblers	137 875	8.3		
Elementary occupations	178 327	10.7		
Armed forces	10 937	0.7		
Unknown occupations	140 045	8.4		

All covariates were updated annually from 2000–2009, except sex and migration background that were included based on the status in 2000.

### Statistical analysis

All analyses were conducted using SAS 9.4 (SAS Institute, Cary, NC, USA). Individuals were followed from year 2000 until the first CHD event, censoring (emigration from Denmark, non-CHD death), or end of follow-up, whichever came first. Using Cox proportional hazard models with calendar time as the underlying time axis we calculated HR and 95% CI for the association between job strain and incident CHD.

We performed three main analyses, depicted in supplementary appendix 2, figure S1. First, we performed a “traditional” analysis with job strain measured at baseline (year 2000) and first CHD event during follow-up (2001–2010) (analysis #1).

Second, we examined the association between number of years exposed to job strain in the five years before start of follow-up (1996–2000) and first CHD event during follow-up (2001–2010). This analysis was restricted to individuals who were employed in all years from 1996–2000 (N=1 353 249) (analysis #2).

Third, we analyzed the association between persistent job strain, as well as job strain onset and removal, and first CHD event during follow-up, ie, the association between changes in job strain from year t-1 to year t (1999–2000, 2000–2001, … 2008–2009) and first CHD event in year t+1 (2001, 2002, … 2010) (analysis #3). Individuals contributed with exposure information in each exposure period until they were removed from the analysis because of a CHD event or censoring. We further coded if an individual moved from job strain or no job strain to non-employment and vice versa or if an individual remained in non-employment.

We incrementally adjusted analyses for sex, age, family type, migration background, health services use, and household disposable income. In analyses #1 and #2, all covariates were treated as time-invariant based on year 2000 values. In analysis #3, sex and migration background were treated as time-invariant, whereas age, family type and household income were treated as time-varying with a one-year time lag between the measurement of the covariates and the measurement of the outcome. Health service use was treated as time-varying, with a two-year time lag between the measurement of the covariate and the measurement of the outcome to ensure that health service use was measured before job strain and, therefore, was not a mediator of the association between job strain and incident CHD.

We conducted four supplementary analyses. First, to examine if associations differed when enhancing exposure contrast, we repeated analysis #1 analyzing job strain categorized into quartiles instead of dichotomized by median split. Second, to examine if associations were different for men and women, we repeated the main analyses separately for men and women. Third, we repeated the main analyses with further adjustment for education. Fourth, we repeated the main analyses conducted separately by educational level.

## Results

### Characteristics of the study population at baseline

Table 1 shows the characteristics of the study population in 2000. Men and women were nearly equally represented and mean age was about 44 years. The most prevalent occupational groups were technicians and associate professionals (19.0%), followed by professionals (15.2%), clerks (11.7%), and service workers and shop and market sales workers (11.5%).

### Job strain in 2000 and incident CHD (analysis #1)

Table 2 shows the association between job strain, measured in 2000, and incident CHD from 2001–2010. Mean time of follow-up was 9.71 years. During 16 117 512 person-years, we identified 24 159 incident cases of CHD (15.0 cases per 10 000 person-years), 11 032 in the no job strain group (13.7 per 10 000 person-years) and 13 127 in the job strain group (16.3 per 10 000 person-years). The HR for comparing individuals with job strain to individuals without job strain was 1.16 (95% CI 1.13–1.19) after adjustment for age, sex, family type, migration background and health service use (model 2) and 1.10 (95% CI 1.07–1.13) after further adjustment for household disposable income (model 3).

**Table 2:** Association between job strain measured in 2000 and incident coronary heart disease (CHD) (2001–2010) among 1 660 150 employees in Denmark (analysis #1). Covariates were measured in 2000 and treated as time-invariant. [HR=hazard ratio; CI=confidence interval]

Job strain at baseline	Person-years	Number of cases	Cases per 10 000 person-years	Model 1 ^a^ HR (95% CI)	Model 2 ^b^ HR (95% CI)	Model 3 ^c^ HR (95% CI)
No job strain	8 045 595	11 032	13.7	1.00	1.00	1.00
Job strain	8 071 917	13 127	16.3	1.17 (1.14–1.20)	1.16 (1.13–1.19)	1.10 (1.07–1.13)

a Adjusted for sex and age.

b Model 1 plus further adjustment for family type, migration background and health service use.

c Model 2 plus further adjustment for household disposable income.

### Years with job strain and incident CHD (analysis #2)

Table 3 shows the association between number of years with job strain from 1996–2000 and incident CHD from 2001–2010. Mean time of follow-up was 9.74 years. This analysis was restricted to individuals who were employed in all years from 1996–2000 (N=1 353 249 providing 13 176 222 person-years). Compared to individuals with no exposure to job strain, there was a higher risk of CHD for those with exposure during 1–2 years (HR 1.21, 95% CI 1.16–1.26), 3–4 years (HR 1.20, 95% CI 1.15–1.26), or all 5 years (HR 1.14, 95% CI 1.11–1.18) in the most adjusted analysis. There was no indication of a dose–response association, ie, after 1–2 years of job strain, the risk did not increase further with increasing number of years with job strain.

**Table 3:** Association between number of years with job strain measured from 1996–2000 and incident coronary heart disease (CHD) from 2001–2010 among 1 353 249 employees in Denmark employed from 1996–2000 (analysis #2). Covariates were measured in 2000 and treated as time-invariant. [HR=hazard ratio; CI=confidence interval].

Years exposed to job strain	Person-years	Number of cases	Cases per 10 000 person-years	Model 1 ^a^ HR (95% CI)	Model 2 ^b^ HR (95% CI)	Model 3 ^c^ HR (95% CI)
0	4 652 463	6360	13.7	1.00	1.00	1.00
1–2	1 803 121	2930	16.2	1.29 (1.23–1.35)	1.28 (1.22–1.34)	1.21 (1.16–1.26)
3–4	2 140 164	3329	15.6	1.31 (1.25–1.36)	1.29 (1.24–1.34)	1.20 (1.15–1.26)
5	4 580 474	7432	16.2	1.25 (1.21–1.29)	1.23 (1.19–1.28)	1.14 (1.11–1.18)

a Adjusted for sex and age.

b Model 1 plus further adjustment for family type, migration background and health service use.

c Model 2 plus further adjustment for household disposable income.

### Persistent job strain, onset of job strain and removal of job strain and incident CHD (analysis #3)

Table 4 shows the association between persistent job strain, onset of job strain, and removal of job strain from year t-1 to year t and incident CHD in year t+1. Compared to the reference group of persistent no job strain, there was a higher risk of incident CHD among individuals with persistent job strain (HR 1.07, 95% CI 1.03–1.10), individuals moving from no job strain to job strain (onset) (HR 1.20, 95% CI 1.12–1.29), and individuals moving from job strain to no job strain (removal) (HR 1.20, 95% CI 1.12–1.28) after adjustment for covariates.

**Table 4:** Association between persistent, onset and removal of job strain measured from 2000–2009 and incident coronary heart disease (CHD) from 2001–2010, among 1 660 150 employees in Denmark with a one-year time lag between exposure and outcome (analysis #3). Age, family type and income were time-varying and measured annually concurrent with job strain. Health service use was time-varying and measured annually one year before job strain. Sex and migration background were time-invariant and measured in 2000. [HR=hazard ratio; CI=confidence interval].

Exposure to job strain from one year to the subsequent year	Person-years	Number of cases	Cases per 10 000 person-years	Model 1 ^a^ HR (95% CI)	Model 2 ^b^ HR (95% CI)	Model 3 ^c^ HR (95% CI)
Persistent no job strain	6 232 572	8071	12.9	1.00	1.00	1.00
Persistent job strain	6 270 275	8272	13.2	1.15 (1.12–1.19)	1.14 (1.11–1.18)	1.07 (1.03–1.10)
No job strain to job strain (onset)	601 343	836	13.9	1.29 (1.20–1.38)	1.27 (1.18–1.37)	1.20 (1.12–1.29)
Job strain to no job strain (removal)	605 352	956	15.8	1.29 (1.20–1.38)	1.27 (1.19–1.36)	1.20 (1.12–1.28)
Job strain to out of employment	316 762	727	23.0	1.55 (1.43–1.67)	1.44 (1.34–1.56)	1.23 (1.14–1.33)
No job strain to out of employment	326 564	787	24.1	1.44 (1.34–1.55)	1.36 (1.26–1.46)	1.19 (1.11–1.29)
Out of employment to job strain	167 302	235	14.0	1.51 (1.32–1.72)	1.41 (1.24–1.60)	1.23 (1.08–1.40)
Out of employment to no job strain	170 912	230	13.5	1.21 (1.06–1.37)	1.15 (1.01–1.31)	1.04 (0.91–1.18)
Persistent out of employment	1 384 460	4045	29.2	1.60 (1.53–1.67)	1.49 (1.43– 1.55)	1.27 (1.21–1.33)

a Adjusted for sex and age.

b Model 1 plus further adjustment for family type, migration background and health service use.

c Model 2 plus further adjustment for household disposable income.

Compared to individuals with persistent no job strain, exiting employment was associated with an increased risk of CHD, both for those exiting from a job with job strain (HR 1.23, 95% CI 1.14–1.33) and those exiting from a job without job strain (HR 1.19, 95% CI 1.11–1.29). Entering employment was associated with a higher risk of CHD if individuals entered a job with job strain (HR 1.23, 95% CI 1.08–1.40), but not if individuals entered a job without job strain (HR 1.04, 95% CI 0.91–1.18).

### Supplementary analyses

To enhance exposure contrast, we repeated analysis #1 with a job strain variable categorized by quartiles instead by median split. Compared to the group with low job strain in 2000, HR for CHD were 1.20 (95% CI 1.15–1.24), 1.18 (95% CI 1.14–1.23) and 1.24 (95% CI 1.19–1.29) for the groups with medium-low, medium-high and high job strain, respectively (appendix 3, table S2).

Repeating the main analyses separately for men and women did not reveal any major differences between the sexes (appendix 4, tables S3–5).

When we repeated the main analyses, while adjusting for education, estimates became attenuated with some estimates (eg, onset of job strain) remaining considerable (appendix 5, tables S6–8). When we conducted analyses separately by educational level, associations between job strain and risk of CHD were strongest for individuals with a high level of education, however CI overlapped (appendix 6, tables S9–11).

## Discussion

In this population-based study of the Danish workforce, persistent and changing exposure to job strain – compared to persistent absence of job strain – was associated with a higher risk of CHD, defined as non-fatal myocardial infarction or CHD mortality. These results suggest that the psychosocial work environment in general, and job strain in particular, may contribute to the etiology of CHD. Using repeated measures of exposure and assessing job strain not by self-report but with a JEM, this study addressed important limitations of most previous studies.

**Figure 1:**
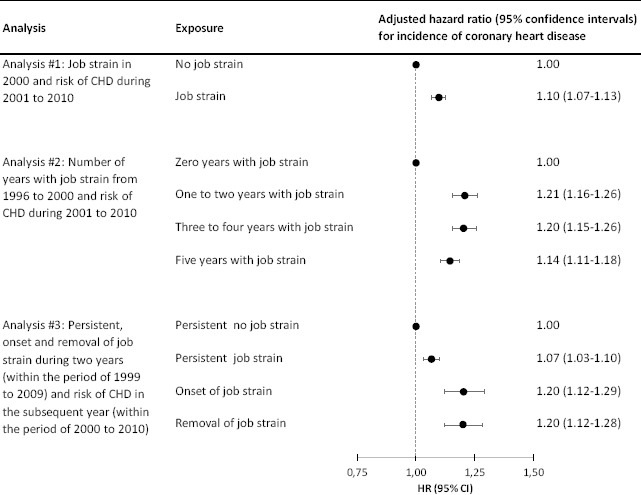
Forest plot summarizing the main results on the association between job strain and incident coronary heart disease (CHD). Hazard ratios are adjusted for sex, age, family type, health service use, and disposable household income.

The main results are summarized in figure 1. Job strain predicted CHD when measured (i) at baseline, (ii) as the number of years with job strain in a five-year period before start of follow-up, (iii) as persistent job strain in two subsequent years, (iv) as onset of job strain, defined as moving from the no job strain category to the job strain category, and (v) as removal of job strain, defined as moving from the job strain category to the no job strain category. We found no clear dose–response patterns between years with job strain and risk of CHD.

### Comparison with previous studies on job strain and CHD

With more than 1.6 million individuals and more than 16 million person-years of follow-up, this is the largest prospective study so far on job strain and risk of CHD. The second-largest study was the IPD-Work consortium analysis that reported a pooled HR of 1.23 in 13 harmonized European cohort studies with 197 473 participants providing 1.49 million person-years (1). Although we used a different methodological approach in JEMPAD compared to IPD-Work, the JEMPAD estimates were either similar to, or only slightly lower than, the estimates from IPD-Work.

To our knowledge, no large-scale study has previously analyzed the association of onset and removal of job strain and risk of CHD. In an analysis of 7253 civil servants from the British Whitehall II cohort, Kivimäki et al (21) examined job strain at two points, three years apart, and reported elevated HR of CHD for job strain score at both phase 1 (HR 1.23, 95% CI 1.10–1.38 per one standard deviation increase) and phase 2 (HR 1.15, 95% CI 1.03–1.29). Changing job strain score from phase 1 to phase 2 was not related to risk of CHD. As these analyses were based on a continuous job strain score, estimates on onset and removal of job strain were not available.

To analyze the association between job strain and risk of CHD using a JEM is not novel. Case–control studies using JEM were the method of choice in the beginning of job strain research in Sweden in the 1980s (22–25). From the mid-1990s, when an increasing number of cohort studies with individual-level job strain exposure data became available (3), JEM analyses of job strain went out of fashion. Recently, there has been a revival of interest in psychosocial job exposure matrices, with the development of new matrices for different psychosocial work environment factors in The Netherlands (26), France (27), Finland (28), Denmark (29, 30), and Australia (31). To our knowledge, the most recently published study on job strain and CHD using a JEM was a study with 6070 Swedish men that reported a HR of 1.31 (95% CI 1.01–1.70) among those exposed to job strain (32). Unlike our study, though, the Swedish study did not examine changes of job strain over time.

### Interpretation

There has been a concern with previous studies that estimates of the association between job strain and CHD may be inflated due to reporting bias, as individuals with undetected pre-clinical CHD might experience and report working conditions as more strenuous than individuals without pre-clinical CHD (33). Because we used a JEM to ascertain job strain, the measurement of the exposure to job strain was not dependent on the individuals’ report of working conditions, and we can rule out that reporting bias has inflated our estimates. However, a weakness of JEM is their inability to detect differences in job strain levels within occupational groups, likely resulting into non-differential exposure misclassification and bias towards an underestimation of associations.

We defined job strain as a dichotomous variable, comparing job strain with no job strain, as this was also the definition used by the IPD-Work consortium (1). Another often used operationalization is the quadrant model, with the four groups of no job strain (low demands and high control), passive work (low demands and low control), active work (high demand and high control), and job strain (high demands and low control). The quadrant model may have yielded different results, and we encourage further research on this model.

To assess job strain in the register data, we used the median split of the predicted probabilities of job strain, resulting in 50% exposed and 50% unexposed and a HR of 1.10 in the analysis on job strain in 2000 and CHD during 2001–2010. When we enhanced exposure contrast by categorizing job strain into quartiles, the HR for the quartile with the highest predicted probability of job strain was 1.24. This result indicates that our estimates may have been conservative and would have been stronger if we had defined job strain with a higher exposure contrast.

There was no indication that chronic job strain over a five-year period was more hazardous than exposure of 1–2 years only during a 5-year period. It is possible that this estimate for chronic job strain was affected by healthy worker selection, with employees remaining in high strain jobs throughout the 5-year period being healthier or more resilient than those changing to lower strain jobs.

That individuals moving from the no job strain to the job strain category had a HR of CHD of 1.20 – compared to individuals remaining in the no job strain category – may provide the strongest argument for the case that there is a causal effect of job strain on risk of CHD. The interpretation of a causal effect is further strengthened by the result that individuals entering employment were –compared to the persistent no job strain group – at higher risk of CHD when they entered a job with job strain but not when they entered a job without job strain.

Removal of job strain was associated with an increased risk of CHD of the same magnitude as onset of job strain when comparing the two groups to persistent no job strain. One explanation may be reverse causation, ie, individuals with pre-clinical CHD moving to jobs without job strain shortly before manifest a clinical CHD event. A similar association has been observed in a study on working hours, where myocardial infarction was more common when the participant either worked long working hours or short hours as a part-timer (34). We tried to account for health selection by adjusting for health-services use, but a residual selection effect might have remained.

Staying persistently out of employment and moving from employment to out of employment were both associated with an increased risk of CHD. This may reflect health selection, ie, that individuals with pre-clinical CHD are no longer able to stay in employment, or a causal effect of unemployment on the risk of CHD (35), or both.

Because of the large sample size, it is possible that small and clinical unimportant estimates may become “statistically significant”. Thus, the interpretation of the estimates should not primarily focus on statistical significance but should focus on the magnitudes of the point estimate and the estimates within the CI. Considering that the HR of CHD associated with onset of job strain (1.20, 95% CI 1.12–1.29) was of about the same magnitude as the HR of cardiovascular disease associated with obesity in the literature (36), we conclude that job strain was associated with a moderate, but clinically important, excessive risk of CHD.

### Strengths and limitations

The strengths of this study are its population-based design, the large study population, the number of cases yielding precise estimates with narrow CI, the register-based outcome ascertainment, and the repeated measure of exposure and confounders. To our knowledge, this is the first study analyzing the association between onset and removal of job strain and risk of CHD. Given the large study population, we were able to conduct analyses separately for men and women, demonstrating that associations were similar in both sexes. Using a JEM to assess job strain ensured that estimates were not affected by reporting bias.

The study also had several important limitations. Because we measured job strain with a JEM, we do not know if the individuals were indeed exposed to job strain but rather only that they worked in a job with a certain exposure probability. Consequently, there may have been non-differential exposure misclassification, likely biasing the estimates towards an underestimation of associations. Exposure misclassification may also have been caused by the fact that we measured job strain not with a standard instrument but with items approximating such an instrument and by selective non-response in the DWECS survey.

We defined persistent job strain as exposure to job strain in two subsequent years. We acknowledge that alternative definitions of persistence, using longer time periods, may have yielded different results.

It is possible that we overestimated the association between job strain and CHD due to residual confounding by socioeconomic position. Some studies have reported a higher prevalence of job strain in employees of low socioeconomic position (37) and it is well documented that low socioeconomic position is strongly associated with a higher risk of CHD in high-income countries (38), including Denmark (8). Consequently, we adjusted the estimates for an indicator of socioeconomic position, ie, household disposable income, and these adjustments resulted in attenuated estimates (as can be seen in tables 2–4 when comparing model 2 with model 3). In contrast to most other studies, we measured socioeconomic position not only at baseline but also during follow-up, increasing precision in the measurement of the potential confounder and allowing us to treat socioeconomic position as a time-varying covariate in some of the analyses. While this is a strength of our study, residual confounding by socioeconomic position remains a possibility and adjustments for additional indicators of socioeconomic position would have strengthened the study. However, we refrained from doing so in the main analyses as the two other measures of socioeconomic position in JEMPAD – occupational status and educational attainment, – were intertwined with our job-group-based measure of job strain. For the sake of completeness, we provided estimates adjusted for education in the supplementary analyses. As expected, the estimates were attenuated, but some remained of a considerable magnitude (eg, onset of job strain). When exploring the association between job strain and CHD across educational levels, we found a tendency for stronger associations among those with a high level of education. Previously, we had reported that the association between low education and cardiovascular morbidity and mortality was attenuated after accounting for both household income and job strain (8). Further studies examining the interplay of socioeconomic position and job strain with regard to risk of CHD are recommended.

We had no information on behavioral risk factors of CHD such as smoking, unhealthy diet or lack of physical activity (36). This can be considered a limitation, as many studies on job strain and CHD have routinely adjusted for these variables (1). However, smoking, diet and lack of physical activity may represent mediators rather than potential confounders for the association between job strain and CHD (39). Adjustment for factors on the causal pathway does not inform about confounding but would rather lead to underestimation of the association between the exposure and the outcome.

There are several other psychosocial work environment conditions that, either alone or in interplay with job strain, may affect coronary health. A recent meta-analysis showed that exposure to either job strain or effort–reward imbalance was associated with a 1.16-fold higher risk of CHD, whereas simultaneous exposure to both job strain and effort–reward imbalance yielded a 1.41-fold higher risk (4). Other recent studies suggest that exposure to job insecurity (2), workplace bullying (5), and workplace violence (5) may also contribute to risk of cardiovascular disease. We plan to address some of these potential risk factors in future analyses in JEMPAD.

### Concluding remarks

Job strain, ascertained by a JEM, was associated with a 7–21% higher risk of CHD in the Danish workforce. This association was similar for men and women and was seen across different types of analyses, including job strain at baseline, number of years with job strain, persistent job strain, and onset of job strain. Removal of job strain in the year preceding the CHD event also yielded a higher risk of CHD, compared to persistent no job strain. One possible explanation for this result could be health selection.

It has been suggested that causal inference from observational epidemiological studies can be strengthened by triangulation, ie, combining results from different approaches with different methodological strengths and limitations, ideally with potential biases that are in opposite directions (40). We suggest that our results have strengthened the evidence that job strain may be a causal factor in the etiology of CHD. Our estimates were similar to the estimate of the IPD-Work consortium analyses that measured job strain not with a JEM but with individual-level data, an approach that was more vulnerable to reporting bias but less vulnerable to non-differential misclassification than the approach taken in this study.

## Supplementary material

Supplementary material

## Protection of research participants

This study complies with the Declaration of Helsinki. No patients were involved. In Denmark, studies that are based on questionnaire and register-data only do not require approval from the National Committee on Health Research Ethics. The Danish Data Protection Agency approved this study through the joint notification of the National Research Centre for the Working Environment (#2015-57-0074). All data are stored in a protected server environment hosted by Statistics Denmark.
