# Supplementary material for: Persistent and changing job strain and risk of coronary heart disease. A population-based cohort study of 1.6 million employees in Denmark
Source: Scand J Work Environ Health. 2020 Mar 23;46(5):498–507. doi: 10.5271/sjweh.3891 (PMC7737794; doi:10.5271/sjweh.3891)
Supplement: Supplementary material [file SJWEH-46-498-S001.pdf]

Persistent and changing job strain and risk of coronary heart disease. A population-based cohort study of 1.6 million employees in Denmark [1]

by Reiner Rugulies, PhD,[2] Elisabeth Framke, PhD, Jeppe Karl Sørensen, MSc, Annemette Coop Svane-Petersen, PhD, Kristina Alexanderson, PhD, Jens Peter Bonde, MD, PhD, Kristin Farrants, PhD, Esben Meulengracht Flachs, PhD, Linda L Magnusson Hanson, PhD, Solja T Nyberg, PhD, Mika Kivimäki, FMedSci, Ida EH Madsen, PhD

1. *Supplementary material*
2. *Correspondence to: Reiner Rugulies, National Research Centre for the Working Environment, Lersø Parkallé 105, DK-2100 Copenhagen, Denmark. [E-mail: rer@nrcwe.dk]*

- **Appendix 1**: Ascertainment of job strain with a job exposure matrix
- **Appendix 2**: Overview of the three main analyses in the study
- **Appendix 3**: Analysis of the association between job strain, defined by quartiles, and risk of CHD
- **Appendix 4**: Analysis of the association between job strain and risk of CHD conducted separately for men and women
- **Appendix 5**: Analysis of the association between job strain and risk of CHD additionally adjusted for education
- **Appendix 6**: Analysis of the association between job strain and risk of CHD conducted separately for educational groups

Appendix 1: Ascertainment of job strain with a job exposure matrix

To ascertain job strain we constructed a job exposure matrix based on information from the Danish Work Environment Cohort Study (DWECS) and assigned the matrix to individuals in the JEMPAD (Job Exposure Matrix Analysis of Psychosocial Factors and Healthy Ageing in Denmark) cohort. DWECS was a survey on working conditions and health conducted in a random sample of employed individuals in Denmark, aged 18 to 64 years (1, 2), first drawn in 1990 and followed-up every fifth year until 2010, with inclusion of additional individuals in each wave. We included DWECS data from the years 2000 (response rate: 75%) (1) and 2005 (response rate 63%) (2). In accordance with previous research on job strain and cardiovascular disease using DWECS data (3-5), we measured job strain by combining three items measuring job demands and five items measuring job control (see **Table S1**). Several of the items originated from the Copenhagen Psychosocial Questionnaire, version I (6).

Respondents were included if they responded to at least two of the three job demands items and to at least three of the five job control items. We calculated scales for job demands and job control with higher scores indicating higher job demands and higher job control, respectively. We defined job strain in DWECS respondents as scoring simultaneously above the median on job demands and below the median on job control, as in previous research (3-5).

We categorized DWECS respondents in job groups according to the DISCO-88 occupational classification system (7). DISCO-88 is the Danish version of the International Standard Classification of Occupations (ISCO) developed by the International Labour Organization (ILO) and was used from 1991 to 2009, when it was replaced with DISCO-08 (8). We used the four-digit level classification and required a minimum of five respondents in DWECS within each job group. Job groups with four or less respondents were collapsed with other similar small job groups at the three-digit or two-digit classification level in the DISCO-88 classification system. This approach was used to avoid developing the job exposure matrix based on job groups with a very small number of respondents, as this would lead to imprecise estimates.

Using the Glimmix procedure in SAS 9.4, we estimated the predicted probabilities of job strain given job group (according to the DISCO-88 classification system), sex, age and year of data collection (2000, 2005). Thus, we constructed a job exposure matrix that was job group-, sex-, age-, and period-specific. After linking the predicted probability for job strain on a yearly basis to the individuals in the JEMPAD cohort, we dichotomized the participants into "no job strain" versus "job strain", based on median split of the distribution of individuals within each year. We chose to use yearly median split rather than absolute cut-off points for the categorization of job strain because there was a rather strong effect of wave of data collection in the data used to estimate the job exposure matrix (DWECS 2000 vs. DWECS 2005). Because exposure was assigned based on predicted values from the year 2000 period-specific job exposure matrix during the years 1996-2003 and predicted values from the year 2005 period-specific job exposure matrix during the years 2004-2009, we could not establish absolute cut-off values for the categorization of job strain that would be meaningful throughout the entire period of exposure assessment.

Participants who were not employed, e.g. due to unemployment or retirement, were assigned to a separate category of not applicable (NA) job strain during their time outside of employment.

To assess the performance of the job exposure matrix measuring job strain, we calculated the area under the curve (AUC) for job strain. The AUC was estimated by comparing the agreement between the job exposure matric values for job strain to the individual-level self-reported job strain in DWECS. The AUC was 0.70, which can be regarded as a fair agreement, considering that AUCs may range from 0.50 (agreement by chance) to 1.00 (perfect agreement). We conclude that the job exposure matrix assessing job strain performed reasonably well in DWECS and could therefore be used for estimating the association between job strain and coronary heart disease in register-based population studies.

Table S1. Scales, items and response options for job strain variable

Scales	Items	Response options
**Job demands**	Do you have to work very fast?	Always; Often; Sometimes; Seldom; Never/hardly ever
	How often do you not have time to complete all your work tasks?	Always; Often; Sometimes; Seldom; Never/hardly ever
	Contradictory demands are placed on you at work?	Correct; From time to time correct; Not correct
**Job control**	Do you have any influence on what you do at work?	Always; Often; Sometimes; Seldom; Never/hardly ever
	Can you use your skills or expertise in your work?	To a very large extent; To a large extent; Somewhat; To a small extent; To a very small extent
	Do you have the possibility of learning new things through your work?	To a very large extent; To a large extent; Somewhat; To a small extent; To a very small extent
	Does your work require you to take the initiative?	To a very large extent; To a large extent; Somewhat; To a small extent; To a very small extent
	Is your work varied?	To a high degree; To some degree; Only to a lesser degree; No, or only to a slight degree

References

1. Burr H, Bjorner JB, Kristensen TS, Tüchsen F, Bach E. Trends in the Danish work environment in 1990-2000 and their associations with labor-force changes. Scand J Work Environ Health. 2003;29(4):270-9.
2. Feveile H, Olsen O, Burr H, Bach E. Danish Work Environment Cohort Study 2005: From idea to sampling design. Statistics in Transition. 2007;8(3):441-58.
3. Fransson EI, Nyberg ST, Heikkilä K, Alfredsson L, De Bacquer D, Batty GD, et al. Comparison of alternative versions of the job demand-control scales in 17 European cohort studies: the IPD-Work consortium. BMC Public Health. 2012;12(1):62.
4. Kivimäki M, Nyberg ST, Batty GD, Fransson EI, Heikkilä K, Alfredsson L, et al. Job strain as a risk factor for coronary heart disease: a collaborative meta-analysis of individual participant data. Lancet. 2012;380(9852):1491-7.
5. Fransson EI, Nyberg ST, Heikkilä K, Alfredsson L, Bjorner JB, Borritz M, et al. Job strain and the risk of stroke: an individual-participant data meta-analysis. Stroke. 2015;46(2):557-9.
6. Kristensen T, Hannerz H, Høgh A, Borg V. The Copenhagen Psychosocial Questionnaire. A tool for the assessment and improvement of the psychosocial work environment. Scand J Work Environ Health. 2005;31(6):438-49.
7. International Labour Organization (ILO). ISCO-88. 2004. Available from: http://www.ilo.org/public/english/bureau/stat/isco/isco88/. (Accessed: 22 May 2019).
8. International Labour Organization. ISCO-08 Structure, index correspondence with ISCO-88. 2016. Available from: http://www.ilo.org/public/english/bureau/stat/isco/isco08/. (Accessed: 22 May 2019).

Appendix 2: Overview of the three main analyses in the study

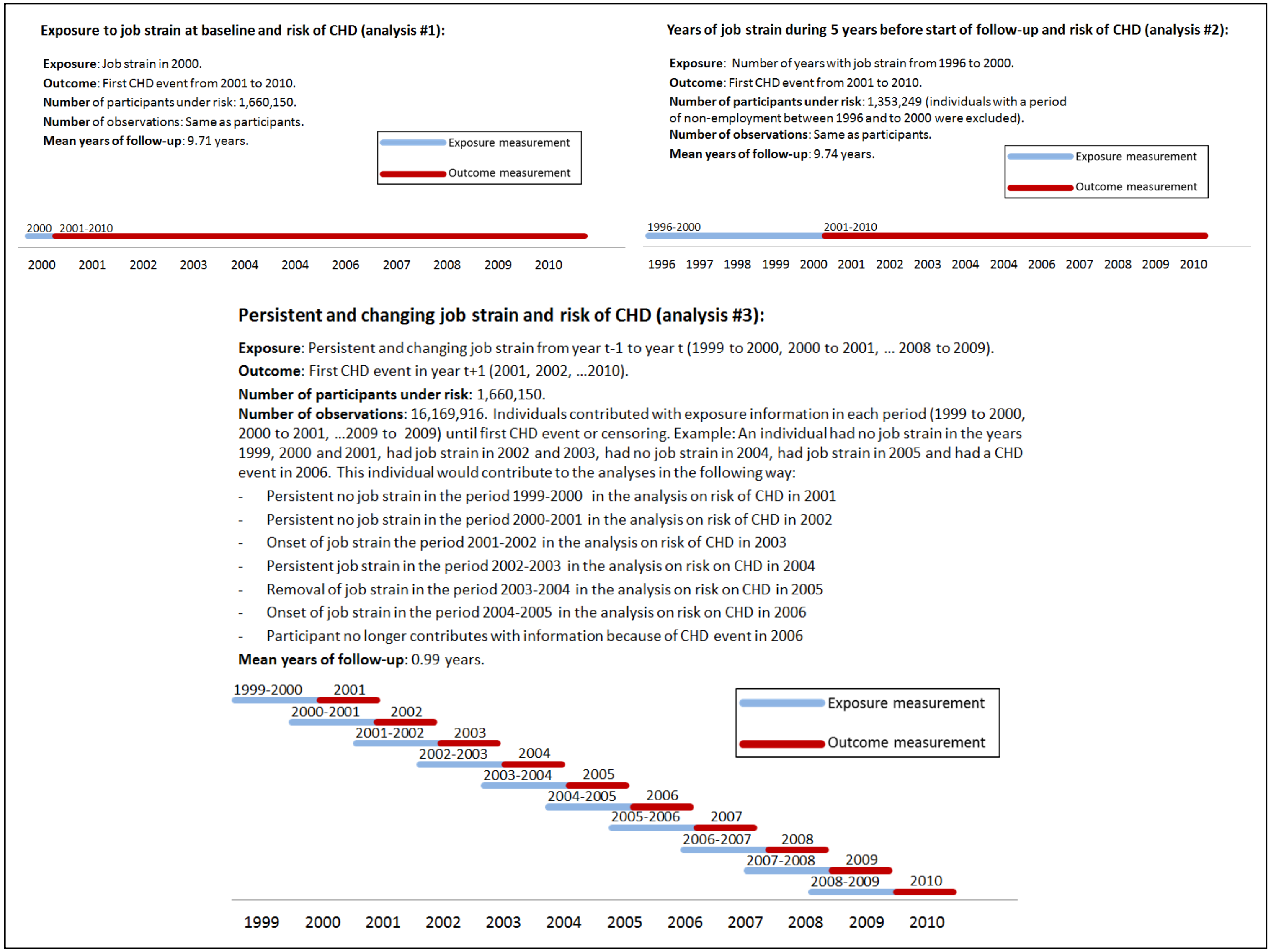

Figure S1: Design of the three main analyses in the study

Appendix 3: Analysis of the association between job strain, defined by quartiles, and risk of CHD

Table S2. Association between job strain, defined by quartiles, measured in 2000 and incident coronary heart disease from 2001 to 2010 among 1,660,150 employees in Denmark

Job strain at baseline	Person-years	Number of cases	Cases per 10,000 person-years	Adjusted HR (95% CI)
**All**				
Low job strain	4,027,420	5,028	12.5	1.00
Medium-low strain	4,018,175	6,004	14.9	1.20 (1.15-1.24)
Medium-high job strain	4,031,494	6,686	16.6	1.18 (1.14-1.23)
High job strain	4,040,423	6,441	15.9	1.24 (1.19-1.29)

HR: Hazard ratio; 95% CI: 95% Confidence interval-
Adjusted for age, family type, migration background, health service use and household disposable income. Covariates were measured in the year 2000 and were treated as time-invariant.

Appendix 4: Analysis of the association between job strain and risk of CHD conducted separately for men and women

Table S3. Association between job strain measured in 2000 and incident coronary heart disease from 2001 to 2010 among 850,999 men and 809,151 women

Job strain at baseline	Person-years	Number of cases	Cases per 10,000 person-years	Adjusted HR (95% CI)
**Men**				
No job strain	3,921,227	8,435	21.5	1.00
Job strain	4,271,418	10,444	24.5	1.10 (1.06-1.13)
**Women**				
No job strain	4,124,368	2,597	6.3	1.00
Job strain	3,800,499	2,683	7.1	1.11 (1.05-1.17)

HR: Hazard ratio; 95% CI: 95% Confidence interval.
Adjusted for age, family type, migration background, health service use and household disposable income. Covariates were measured in the year 2000 and were treated as time-invariant.

Table S4. Association between number of years with job strain measured from 1996 to 2000 and incident coronary heart disease from 2001 to 2010 among 718,906 men and 634,343 women employed throughout the years 1996 to 2000

Five-year exposure to job strain	Person-years	Number of cases	Cases per 10,000 person-years	Adjusted HR (95% CI)
**Men**				
Zero years with job strain	2,337,612	4,900	21.0	1.00
One to two years with job strain	1,145,571	2,563	22.4	1.22 (1.16-1.28)
Three to four years with job strain	1,176,730	2,704	23.0	1.22 (1.16-1.28)
Five years with job strain	2,291,559	5,749	25.1	1.15 (1.10-1.20)
**Women**				
Zero years with job strain	2,314,851	1,460	6.3	1.00
One to two years with job strain	657,551	367	5.6	1.15 (1.03-1.29)
Three to four years with job strain	963,434	625	6.5	1.16 (1.06-1.28)
Five years with job strain	2,288,915	1,683	7.4	1.14 (1.06-1.22)

HR: Hazard ratio; 95% CI: 95% Confidence interval.
Adjusted for age, family type, migration background, health service use and household disposable income. Covariates were measured in the year 2000 and were treated as time-invariant.

Table S5. Association between persistent, onset and removal of job strain measured from 2000 to 2009 and incident coronary heart disease from 2001 to 2010 among 850,999 men and 809,151 women with a one-year time lag between exposure and outcome

Exposure to job strain from one year to the subsequent year	Person-years	Number of cases	Cases per 10,000 person-years	Adjusted HR (95% CI)
**Men**				
Persistent no job strain	3,208,901	6,455	20.1	1.00
Persistent job strain	3,143,688	6,476	20.6	1.05 (1.01-1.09)
No job strain to job strain (onset)	334,377	689	20.6	1.18 (1.09-1.28)
Job strain to no job strain (removal)	353,981	797	22.5	1.18 (1.10-1.27)
Job strain to out of employment	152,280	561	36.8	1.21 (1.11-1.33)
No job strain to out of employment	164,182	598	36.4	1.15 (1.05-1.25)
Out of employment to job strain	72,427	175	24.2	1.20 (1.03-1.40)
Out of employment to no job strain	87,698	188	21.4	1.04 (0.90-1.20)
Persistent out of employment	653,339	2,940	45.0	1.22 (1.16-1.28)
**Women**				
Persistent no job strain	3,023,671	1,616	5.3	1.00
Persistent job strain	3,126,587	1,796	5.7	1.13 (1.05-1.21)
No job strain to job strain (onset)	266,966	147	5.5	1.28 (1.08-1.51)
Job strain to no job strain (removal)	251,371	159	6.3	1.26 (1.07-1.48)
Job strain to out of employment	164,482	166	10.1	1.29 (1.09-1.51)
No job strain to out of employment	162,382	189	11.6	1.39 (1.19-1.62)
Out of employment to job strain	94,876	60	6.3	1.31 (1.01-1.69)
Out of employment to no job strain	83,214	42	5.0	1.03 (0.76-1.40)
Persistent out of employment	731,121	1,105	15.1	1.45 (1.32-1.59)

HR: Hazard ratio; 95% CI: 95% Confidence interval.

Adjusted for age, family type, migration background, health service use and household disposable income. The covariates age, family type and income were time-varying and were measured annually concurrent with job strain. The covariate health service use was time-varying and was measured annually one year before job strain was measured. The covariant migration background was time-invariant and was measured in the year 2000.

Appendix 5: Analysis of the association between job strain and risk of CHD additionally adjusted for education

Table S6. Association between job strain measured in 2000 and incident coronary heart disease from 2001 to 2010 among 1,660,150 employees in Denmark, additionally adjusted for education at baseline

Job strain at baseline	Person-years	Number of cases	Cases per 10,000 person-years	Adjusted HR (95% CI)
No job strain	8,045,595	11,032	13.7	1.00
Job strain	8,071,917	13,127	16.3	1.00 (0.98-1.03)

HR: Hazard ratio; 95% CI: 95% Confidence interval.
Adjusted for sex, age, family type, migration background, health service use, household disposable income and education.
Covariates were measured in the year 2000 and were treated as time-invariant.

Table S7. Association between number of years with job strain measured from 1996 to 2000 and incident coronary heart disease from 2001 to 2010 among 1,353,249 employees in Denmark employed throughout the years 1996 to 2000, additionally adjusted for education at baseline

Five-year exposure to job strain	Person-years	Number of cases	Cases per 10,000 person-years	Adjusted HR (95% CI)
Zero years with job strain	4,652,463	6,360	13.7	1.00
One to two years with job strain	1,803,121	2,930	16.2	1.08 (1.04-1.13)
Three to four years with job strain	2,140,164	3,329	15.6	1.07 (1.03-1.12)
Five years with job strain	4,580,474	7,432	16.2	1.02 (0.98-1.06)

HR: Hazard ratio; 95% CI: 95% Confidence interval.
Adjusted for sex age, family type, migration background, health service use, household disposable income and education.
Covariates were measured in the year 2000 and were treated as time-invariant.

Table S8. Association between persistent, onset and removal of job strain measured from 2000 to 2009 and incident coronary heart disease from 2001 to 2010, among 1,660,150 employees in Denmark with a one-year time lag between exposure and outcome, additionally adjusted for education at baseline

Exposure to job strain from one year to the subsequent year	Person-years	Number of cases	Cases per 10,000 person-years	Adjusted HR (95% CI)
Persistent no job strain	6,232,572	8,071	12.9	1.00
Persistent job strain	6,270,275	8,272	13.2	0.98 (0.95-1.01)
No job strain to job strain (onset)	601,343	836	13.9	1.11 (1.03-1.19)
Job strain to no job strain (removal)	605,352	956	15.8	1.11 (1.03-1.18)
Job strain to out of employment	316,762	727	23.0	1.14 (1.05-1.23)
No job strain to out of employment	326,564	787	24.1	1.17 (1.09-1.26)
Out of employment to job strain	167,302	235	14.0	1.14 (1.00-1.30)
Out of employment to no job strain	170,912	230	13.5	1.01 (0.88-1.15)
Persistent out of employment	1,384,460	4,045	29.2	1.20 (1.15-1.26)

HR: Hazard ratio; 95% CI: 95% Confidence interval.

Adjusted for sex, age, family type, migration background, health service use, household disposable income and education.

The covariates age, family type, income and education were time-varying and were measured annually concurrent with job strain. The covariate health service use was time-varying and was measured annually one year before job strain was measured. The covariates sex and migration background were time-invariant and were measured in the year 2000.

Appendix 6: Analysis of the association between job strain and risk of CHD conducted separately for education groups

Table S9. Association between job strain measured in 2000 and incident coronary heart disease from 2001 to 2010 among 1,660,150 employees in Denmark by educational level at baseline

Job strain at baseline	Person-years	Number of cases	Cases per 10,000 person-years	Adjusted HR (95% CI)
**Low education**				
No job strain	1,427,985	2,863	20.0	1.00
Job strain	2,315,358	4,783	20.7	0.96 (0.91-1.00)
**Medium education**				
No job strain	3,037,071	4,517	14.9	1.00
Job strain	4,374,273	6,925	15.8	1.01 (0.97-1.05)
**High education**				
No job strain	3,498,922	3,501	10.0	1.00
Job strain	1,268,159	1,129	8.9	1.06 (0.99-1.13)

HR: Hazard ratio; 95% CI: 95% Confidence interval.
Adjusted for sex, age, family type, migration background, health service use and household disposable income.
Covariates were measured in the year 2000 and were treated as time-invariant.

Table S10. Association between number of years with job strain measured from 1996 to 2000 and incident coronary heart disease from 2001 to 2010 among 1,353,249 employees in Denmark employed throughout the years 1996 to 2000 by educational level at baseline

Five-year exposure to job strain	Person-years	Number of cases	Cases per 10,000 person-years	Adjusted HR (95% CI)
**Low education**				
Zero years with job strain	661,348	1,272	19.2	1.00
One to two years with job strain	462,174	1,061	23.0	1.02 (0.94-1.11)
Three to four years with job strain	562,946	1,188	21.1	1.03 (0.95-1.11)
Five years with job strain	1,254,953	2,549	20.3	0.99 (0.92-1.05)
**Medium education**				
Zero years with job strain	1,424,615	2,275	16.0	1.00
One to two years with job strain	899,247	1,425	15.8	1.11 (1.04-1.19)
Three to four years with job strain	1,200,057	1,767	14.7	1.09 (1.03-1.16)
Five years with job strain	2,667,050	4,184	15.7	1.03 (0.97-1.08)
**High education**				
Zero years with job strain	2,533,565	2,745	10.8	1.00
One to two years with job strain	425,189	389	9.1	1.13 (1.02-1.26)
Three to four years with job strain	354,198	322	9.1	1.09 (0.97-1.23)
Five years with job strain	609,077	558	9.2	1.03 (0.94-1.13)

HR: Hazard ratio; 95% CI: 95% Confidence interval.

Adjusted for sex, age, family type, migration background, health service use and household disposable income.

Covariates were measured in the year 2000 and were treated as time-invariant.

Table S11. Association between persistent, onset and removal of job strain measured from 2000 to 2009 and incident coronary heart disease from 2001 to 2010, among 1,660,150 employees in Denmark with a one-year time lag between exposure and outcome by educational level at baseline

Exposure to job strain from one year to the subsequent year	Person-years	Number of cases	Cases per 10,000 person-years	Adjusted HR (95% CI)
**Low education**				
Persistent no job strain	938,644	1,839	19.6	1.00
Persistent job strain	1,601,852	2,785	17.4	0.99 (0.93-1.05)
No job strain to job strain (onset)	157,988	293	18.5	1.08 (0.96-1.22)
Job strain to no job strain (removal)	160,117	312	19.5	1.02 (0.91-1.15)
Job strain to out of employment	84,367	257	30.5	1.18 (1.03-1.35)
No job strain to out of employment	110,492	287	26.0	1.13 (1.00-1.28)
Out of employment to job strain	41,076	66	16.1	0.93 (0.72-1.18)
Out of employment to no job strain	55,669	79	14.2	0.99 (0.79-1.24)
Persistent out of employment	453,118	1,592	35.1	1.24 (1.14-1.34)
**Medium education**				
Persistent no job strain	2,200,467	3,219	14.6	1.00
Persistent job strain	3,570,306	4,629	13.0	0.96 (0.91-1.00)
No job strain to job strain (onset)	301,891	404	13.4	1.07 (0.96-1.18)
Job strain to no job strain (removal)	302,737	479	15.8	1.09 (0.99-1.20)
Job strain to out of employment	130,307	329	25.2	1.16 (1.03-1.30)
No job strain to out of employment	161,855	364	22.5	1.10 (0.98-1.23)
Out of employment to job strain	68,420	99	14.5	1.00 (0.82-1.22)
Out of employment to no job strain	82,646	131	15.9	1.25 (1.05-1.50)
Persistent out of employment	616,724	1,821	29.5	1.19 (1.11-1.28)
**High education**				
Persistent no job strain	3,046,515	2,868	9.4	1.00
Persistent job strain	1,031,492	697	6.8	0.94 (0.87-1.02)
No job strain to job strain (onset)	134,583	116	8.6	1.22 (1.01-1.47)
Job strain to no job strain (removal)	135,302	146	10.8	1.36 (1.15-1.61)
Job strain to out of employment	106,796	189	17.7	1.14 (0.98-1.33)
No job strain to out of employment	38,313	61	15.9	1.31 (1.01-1.69)
Out of employment to job strain	57,946	61	10.5	1.13 (0.88-1.46)
Out of employment to no job strain	24,992	21	8.4	1.03 (0.66-1.60)
Persistent out of employment	290,758	564	19.4	1.12 (1.01-1.24)

HR: Hazard ratio; 95% CI: 95% Confidence interval.
Adjusted for sex, age, family type, migration background, health service use and household disposable income.
The covariates age, family type and income were time-varying and were measured annually concurrent with job strain. The covariate health service use was time-varying and was measured annually one year before job strain was measured. The covariates sex and migration background were time-invariant and were measured in the year 2000.
